# Kinematics of the Coordination of Pointing during Locomotion

**DOI:** 10.1371/journal.pone.0079555

**Published:** 2013-11-19

**Authors:** Enrico Chiovetto, Martin A. Giese

**Affiliations:** Section for Computational Sensomotorics, Department of Cognitive Neurology, Hertie Institute for Clinical Brain Research, Centre for Integrative Neuroscience, University Clinic Tübingen, Tübingen, Germany; VU University Amsterdam, The Netherlands

## Abstract

In natural motor behaviour arm movements, such as pointing or reaching, often need to be coordinated with locomotion. The underlying coordination patterns are largely unexplored, and require the integration of both rhythmic and discrete movement primitives. For the systematic and controlled study of such coordination patterns we have developed a paradigm that combines locomotion on a treadmill with time-controlled pointing to targets in the three-dimensional space, exploiting a virtual reality setup. Participants had to walk at a constant velocity on a treadmill. Synchronized with specific foot events, visual target stimuli were presented that appeared at different spatial locations in front of them. Participants were asked to reach these stimuli within a short time interval after a “go” signal. We analysed the variability patterns of the most relevant joint angles, as well as the time coupling between the time of pointing and different critical timing events in the foot movements. In addition, we applied a new technique for the extraction of movement primitives from kinematic data based on anechoic demixing. We found a modification of the walking pattern as consequence of the arm movement, as well as a modulation of the duration of the reaching movement in dependence of specific foot events. The extraction of kinematic movement primitives from the joint angle trajectories exploiting the new algorithm revealed the existence of two distinct main components accounting, respectively, for the rhythmic and discrete components of the coordinated movement pattern. Summarizing, our study shows a reciprocal pattern of influences between the coordination patterns of reaching and walking. This pattern might be explained by the dynamic interactions between central pattern generators that initiate rhythmic and discrete movements of the lower and upper limbs, and biomechanical factors such as the dynamic gait stability.

## Introduction

In everyday life people accomplish many different motor behaviours that require the coordination of arm movements and locomotion, such as reaching during walking. Although many scientific studies have focused on investigating separately these two motor tasks, surprisingly little research has addressed their interaction and the underlying coordination patterns. We are only aware of a few studies addressing related issues: Georgopoulos and Grillner [1] proposed that reaching and locomotion might share the same control mechanisms. The authors speculated that reaching may have an evolutionary origin, and that it may have evolved from the same neural substrates underling locomotion. Other studies [2], [3] focused on the behavioural aspects of the execution of reaching while walking, and found that subjects tended to exhibit an ipsilateral coupling between the grasping hand and the corresponding foot, potentially to ensure biomechanical stability (different from the usual contralateral extension of upper and lower limbs that is observed during human walking). Other research [4], [5], [6], [7], [8] focused instead on the effects caused by changes of the features characterizing the reaching (such as the shape or the position of the object to reach) during locomotion, showing that almost always subjects had to adjust their gait to meet the demands of the reaching task. Although all these works provided interesting behavioural findings, they left however many questions unanswered and no comprehensive kinematic study has been presented so far that investigates such coordination patterns between locomotion (rhythmic task) and arm reaching (discrete task) in a highly controlled task. To fill this gap we have developed a paradigm that combines locomotion on a treadmill with controlled pointing to targets in the three-dimensional space, exploiting an experimental setup that combines online motion capture and stereoscopic target presentation in Virtual Reality. Participants had to walk at a constant velocity on a treadmill. Synchronized with the heel strike or lift of the toe of either the left or right foot, visual target stimuli were presented that appeared at five different spatial locations in front of them. Participants were asked to reach these stimuli within a short time interval after a “go” signal. By specifying different goal positions and timings of their appearance we required the participants to realize different types of coordinative couplings between the arm and the leg movements, at the same time motion-capturing their body and arm movements. We analysed the variability patterns of the most relevant joint angles, as well as the time coupling between the pointing and the different critical events in the foot movements. In addition, we applied a new technique for the extraction of movement primitives by anechoic demixing in order to identify components in the kinematic data that might be associated with control primitives.

In general, one might speculate about possible outcomes of this experiment, and different hypotheses about the structure of the coordination patterns between reaching and walking could be formulated. A first hypothesis is that both tasks might be controlled simultaneously by exploiting a single coordination strategy. This would represent an efficient solution in terms of the degrees-of-freedom problem [9], [10], [11], [12], [13], since the dimensionality of the relevant controller space would be minimized. However, such a strict coupling of the two behaviours would potentially reduce the flexibility of the realizable behaviours. Alternatively, both tasks might be controlled completely separately. This would then maximize the flexibility, since then both behaviours could be combined in arbitrary ways. However, such separate control might be suboptimal in terms of the dynamic stability of the resulting coordination patterns, since especially for extreme upper-body movements the gait pattern might have to be modified to prevent falling. The hypothesis of the separate control of walking and reaching would be compatible with previous experiments showing different task-specific coordination strategies for reaching and postural tasks during whole-body pointing movements to a target, starting from a standing posture [14], [15], [16], [17]. A third hypothesis would be a flexible architecture with loosely coupled control strategies for walking and reaching. This would still allow for dynamic couplings between both control strategies, if required, but would maintain a modularity of the underlying controller.

In agreement with the last hypothesis we found a modular organization relying on two distinct main kinematic components, accounting for the rhythmic and discrete parts of the movements. Different from strictly hierarchical organizations of the controller architecture, where for example (dynamically stable) walking is controlled with highest priority and provides input to the control of reaching, or vice versa, we found a mutual interaction between locomotion and reaching, i.e. each task to some extent modulates the other one. The fact that we find a strong temporal coupling between kinematic hand and foot events suggests however that walking and reaching seem to be integrated in a coherent common control strategy. These results are interesting because they suggest that such a mutual interaction might result from the interplay between central pattern generator networks (CPGs) involved in upper and lower limb control with voluntary motor commands descending from supraspinal areas, and biomechanical factors, such as the need to maintain dynamical stability during locomotion.

## Methods

### Subjects

Ten healthy right-handed subjects participated to the experiment, 8 males, 2 female, ages 24±5 years (mean ± standard deviation), mass 69±9 kg, height 1.75±0.08 m. All participants were in good health condition and had no previous history of neuromuscular disease. The experiment conformed to the declaration of Helsinki and written informed consent was obtained from all the participants according to the protocol of the local ethical committee (Ethik-kommission an der medizischen Fakultät der Eberhard-Karls-Universität und am Universitätsklinikum Tübingen). The ethic committee had approved this study in advance.

### Apparatus

**Figure 1:**
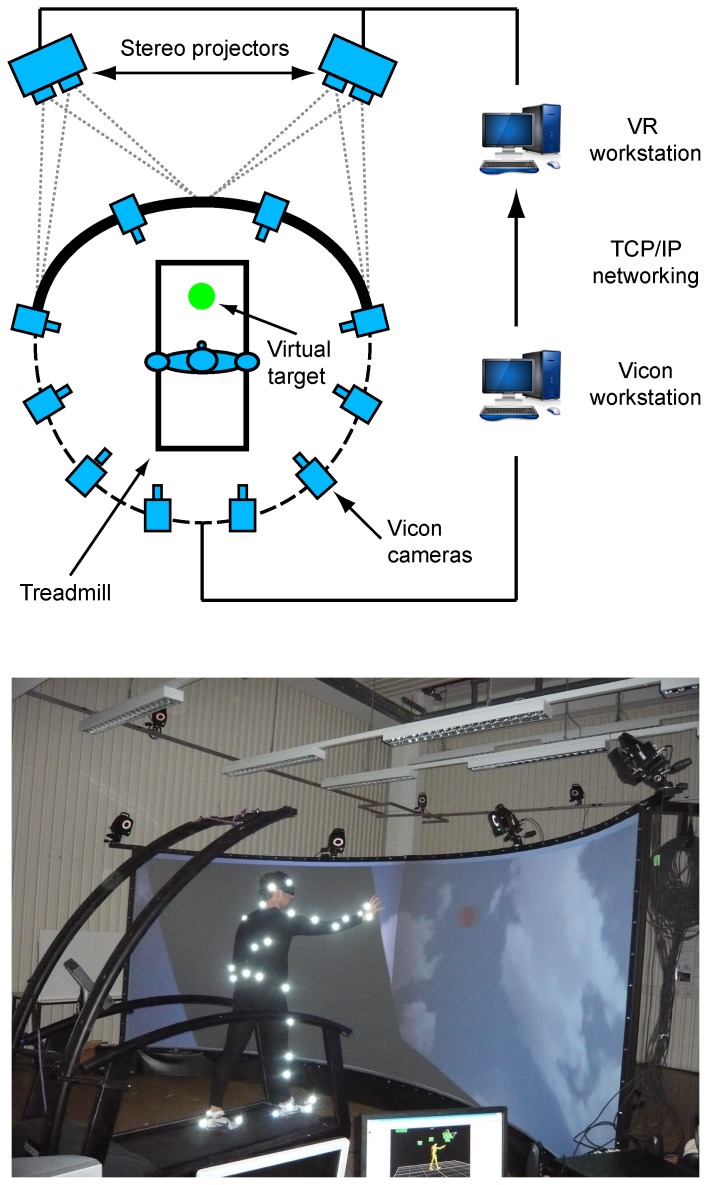
Experimental setup for the study of the coordination of reaching and walking movements. The setup combines motion capture on a treadmill with a virtual reality setup that presents target objects with a precise spatio-temporal relationship to the specific time points of the foot movement (foot events) and the actual position of the participants. Four projectors (two for each half the spherical screen) allow to display virtual objects (spheres, indicated in green on the left side of the figure) in the three-dimensional space in front of the participants, who are walking at a constant speed on a treadmill. Forty-two reflective markers are attached to the participants, and their spatial positions are recorded by an infrared motion capture system (Vicon). The Vicon workstation communicates with the controller of the virtual reality system through the TCP/IP protocol.

The experimental apparatus consisted of a motion capture system, a virtual reality projection system and a treadmill (figure 1). Online motion capture was performed using a Vicon (Oxford, UK) optoelectronic movement recording system with 10 infrared cameras, which recorded the three-dimensional positions of spherical reflective markers (2.5 cm diameter) with spatial error below 1.5 mm. The 42 markers were attached with double-sided adhesive tape to tight clothing, worn by the participants. Marker placement was defined by the Vicon’s PlugInGait marker set. Commercial Vicon software was used to reconstruct and label the markers, and to interpolate short missing parts of the trajectories. Sampling rate was set at 120 Hz. The virtual reality system consisted of 2 pairs of 3D stereo projectors (NEC NP1150, native resolution 1024×768 ppi, horizontal refresh frequency range 15–108 kHz, vertical refresh frequency range 48–120 Hz, modified for stereographic projection by Infitec, Ulm, Germany). Each pair of projectors was covering one half of a big circular screen located in front of the treadmill (either the left or the right one). The motion capture and projection system were controlled by two workstations which were interconnected via a TCP/IP network. This made it possible to control the online generation of 3D stimuli by the actual motion capture data that was processed in real time. Using this setup, we were able to present visual stimuli exactly synchronized with specific foot events (heel strike or toe lift-off of either the right or left foot), which were determined online automatically. The algorithm for the automatic detection of foot events was based on the determination of sign changes of the vertical velocity. This algorithm was suitable for the detection of all four types of foot events. The accuracy of this algorithm was validated by comparing the automatically detected event times with the times obtained by a careful off-line analysis of the motion capture data by visual inspection. The time difference between the automatically detected events and this ground-truth data was on average 22±33 ms across all foot step events. This level of accuracy seemed to justify the use of the automatic detection results instead of a tedious manual analysis of the trials by the inspection of the motion trajectories.

Custom-made software was used to integrate the VR and the Vicon systems, and to generate a geometrically correct stereoscopic projection on the spherical screen.

### Experimental Protocol

Participants were asked to walk at a constant velocity on the treadmill. Synchronized with the heel strike or toe lift-off of either the right or left foot (RHS = Right Heel Strike, RTL = Right Toe Lift-off, LHS = Left Heel Strike, LTL = Left Toe Lift-off), visual target stimuli were presented that appeared at five different spatial locations in front of them. Each stimulus consisted of a virtual ball of 20 cm of diameter. The five targets were located respectively at the vertices and at the centre of an imaginary square lying on a vertical plane parallel to the subjects’ coronal plane. The distance of the vertical plane from the subject corresponded at 115% of the subject’s arm length. Before the start of the recordings, the whole peripersonal space of the subject had first to be calibrated by learning of a nonlinear function that compensated for nonlinear distortions introduced by the geometrical projection errors of the 3D projection system and the semicircular screen. For each participant, the spatial positions of two vertices of the top horizontal side of the square were determined in the following way: the participant was presented with two virtual balls, located at two different horizontally displaced reachable positions on the virtual plane. If the participant was still able to reach these positions with his right hand the virtual balls were located further upwards, left or right, in steps of 5 cm, until the participant almost could not reach them anymore. The other two vertices of the plane were then placed in a way so that they had the same distance from the subject’s frontal and sagittal plane. The distance of the balls from the subject’s coronal plane remained constant during the procedure, and balls remained in the same vertical plane. We made sure that the most extreme target positions exceeded as much as possible the outer boundaries of the subjects’ normal reaching range. In this way the pointing-during-walking task was made as demanding as possible, since (for biomechanical reasons) we did not expect pointing within the comfortably reachable part of the peripersonal space to require specific coordination with walking. By making the motor tasks challenging instead, we aimed to push subjects to realize a wider range of coordination strategies that otherwise would not be necessary to comply with the task.

While continuously walking at a fixed speed (0.8 m/s), the virtual target appeared in one of the five possible positions, triggered by one of the four foot events, which were detected in real time. After the trigger event the colour of the target ball turned red and the motion recoding started. After one single gait cycle, at the occurrence of the same foot event, the target turned yellow in order to notify the subject to get ready for the reaching. After a further gait cycle, when the same foot even occurred again, the target turned green and the participant had to reach with the right hand the virtual ball within a short time interval (duration 0.6 s). The choice of setting the reaching time window 0.6 s long was based on neurophysiological and mechanical considerations, and on a pilot experiment testing different durations. The duration had to be above the sum of the times for the processing of the visual stimulus (colour change), the movement planning time, and the time for sending out the motor command to the muscles, and the time-delay created by the inertia of the musculoskeletal system. During pilot experiments we tested participants’ performances for different durations of the interval and found that for time intervals below 0.6 s the task became too difficult. With the chosen gait velocity the interval duration of 0.6 s resulted in about 25% of invalid reaching movements.

The recording stopped three gait cycles after that the target had turned green. If the target had not been reached within this maximum trial duration the trial was repeated, while otherwise another trial followed. Participants were instructed to reach the balls with the right index finger, although they were told that the precision of the reaching was not crucial for the experiment. This reaching instruction allowed simultaneously to drive the hand close to the targets, and to minimize the variability of the manipulation patterns that would emerge otherwise (such as grasping with different grips, or punching). If at the time of the “go” signal the hand was too close to the virtual target (<30 cm) the trial was discarded and repeated. If, conversely, the pointing was valid and the target was reached within 0.6 s after the occurrence of “go” signal the virtual ball exploded in order to give the participant feedback about the successful pointing. Five successful trials were collected for each of the 20 different experimental conditions (4 foot events by 5 target locations), resulting in a total of 100 trials per subject. The order of the foot events and the target position during each trial was chosen randomly. During each trial the system stored the critical parameters into a log file. Among these parameters were the type of foot event triggering the “go” signal in that trial, the coordinates defining the spatial position of the virtual target displayed in that trial, the time at which the motion capture system detected the foot event, and the time at which the target was reached (i.e. the time at which the hand had a distance from the centre of the virtual ball that was smaller than 5 cm). The walking velocity was chosen based on previous experience in pilot experiments, where participants reported this speed as a comfortable pace for walking. Before starting the data collection, participants were requested to walk freely for several minutes on the treadmill in order to familiarize them with the walking situation. All participants reported at the end that during the walk they felt as comfortable as during natural walking. The experiment was run in complete darkness, with the exception of the light provided by the VR system projecting the stimulus on the screen. None of the participants reported feeling uncomfortable or impaired in the execution of the task for this level of illumination. For safety reasons, the treadmill was equipped with two lateral bars (see picture in figure 1). After the initial walking training phase subjects could maintain their balance on the treadmill very well, so that the lateral bars during the experiment were used only extremely rarely. The fourteen trials in which participants touched the hand-rails were excluded from the analysis.

### Data Analysis

Kinematic data were analysed off-line with customized software written in Matlab (Mathworks, MA). We focused the analysis on a time window that was two gait cycles long. After manual interpolation of missing marker trajectories via commercial Vicon software, only the time intervals of the trajectories from the last lift off of the right toe preceding or coinciding with the “go” signal and the next two gait cycles were considered. One gait cycle was defined as the time between two successive lifts of the same toe. For each trial we computed several parameters. The mean walking velocity was defined as the average, over time, of the difference between the velocity of the belt movement of the treadmill and the velocity along the walking direction of the reflective marker applied on the subject’s right iliac crest. For each gait cycle we computed also its duration and the duration of the stance phase (i.e. the time interval between a foot strike and the successive lift-off), as percentage of the whole gait cycle duration. To define the duration of reaching we considered the temporal evolution of the spatial distance of the marker placed on the dorsum of the subject’s right hand (between the most distal ends of the third and fourth metacarpal bones) and the origin of the world frame of reference, which coincided with the centre of the horizontal surface of the treadmill. This distance changed in an oscillatory fashion and was maximal (in absolute value) at the time at which the target was reached. The duration of reaching was defined as the time interval between successive peaks of this distance. Reach duration was also expressed as percentage of the average gait cycle duration (computed over the two gait cycles within each trial). We also computed the peak velocity of the hand.

Joint angles were computed by approximating the marker positions with a hierarchical kinematic body model (skeleton) with 17 joints (head, neck, spine, and right and left clavicle, shoulder, elbow, wrist, hip, knee and ankle). Coordinate systems were attached to each rigid segment of this skeleton. The rotations between adjacent coordinate systems along this skeleton were characterized by Euler angles, the pelvis coordinate system serving as the origin of the kinematic chain. At the end, all trajectories of the markers and the computed joint angles were resampled with 350 time points and smoothed by spline interpolation. For each participant and trial we computed the time intervals between the “go” signal and time of pointing, as well as the time intervals between the time of pointing and the next four foot step events. To minimize estimation errors, we determined these time intervals in an off-line analysis, by determining the points of maximum hand distance from the origin, taking the whole trajectory into account. Also the times of strike and lift-off of the feet were determined offline by inspection of the trajectories of the heel and toe markers.

We computed the variability (standard deviation) of the time of occurrence of the peaks of amplitude for the most relevant joint angles (specifically those relative to right and left knee and the right shoulder flexion and extension). These angles were the most representative ones for the two main sub-tasks to be accomplished, walking and pointing.

### Dimensionality Reduction Analysis

Dimensionality reduction techniques have become a common tool in the last years in motor control to study the modular organization of movements in both electromyographic [15], [16], [18], [19], [20] and kinematic [17], [21], [22] data. It seems thus as a useful approach for deriving signatures of control modules from the very high-dimensional kinematic data derived from the full-body movements in our task. In previous work on kinematic data the application of principle components analysis (PCA, see [23]) allowed to approximate complex whole-body movements by superpositions of a relatively small number of task-dependent kinematic components, which were interpreted as signatures of an underlying modular control architecture. Kaminski and others [14], [17] showed, for instance, that the identified kinematic components were associated with either postural or reaching components of the task. PCA provided, moreover, additional information about the flexible way how such components were combined in order to accomplish the relevant motor goal. Inspired by these previous investigations, we applied dimensionality reduction algorithms to our data characterizing the coordination of walking and reaching. Besides classical PCA, we also applied a new version of an algorithm for anechoic demixing that allows for temporal shifts in the generative model that reconstructs the signals from the extracted invariant components. We have shown in previous works [24], [25] that this class of mixture models results in very compact representations of kinematic data from complex body movements, often requiring less than half of the number of components than required by PCA models for the same accuracy of approximation.

We present in this paper a new algorithm for anechoic demixing, which is more efficient for problems in motor control than the other algorithms that we have tested. The new technique exploits the fact that the relevant trajectories are always smooth and can be approximated well by anechoic mixtures of smooth signals [22], [26]. Exploiting the smoothness and introducing an appropriate prior for the recovered source functions, we obtain a more efficient and stable demixing algorithm than more general methods that generate estimates assuming more general function classes that are not low-pass-bounded [24]. The core of the method results in an EM-like optimization of the parameters of the following general anechoic mixing model
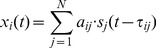
(1)were 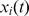 and 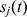 indicate, respectively, the values of the *i-th* joint angle and of the *j-th* temporal component (source) 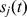 at the time instant *t*. The term anechoic is derived from acoustics, where usually equation (1) is used to describe an acoustic signal as the results of the superposition of multiple and delayed sources of sound in a room without acoustic reflexions. In the following, we will refer to this method as Fourier-based Anechoic Demixing Algorithm, (FADA). Taking into account that the signals are band-limited the temporal signals 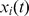 and 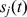 can be approximated by their Fourier expansions:



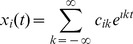
(2)

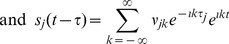
(3)where 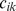 and 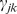 are complex constants (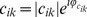
and 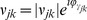). 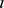 indicates the imaginary unit. Exploiting the assumed smoothness of the signals, we can approximate the signals by the truncated Fourier series



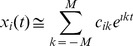
(4)

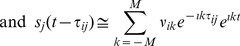
(5)



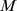
 being a positive integer which is determined by Shannon’s theorem according to the limit frequency of the signals. Substituting (4) and (5) in (1), and assuming uncorrelatedness of the sources 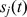, the computation of the both sides of equation (1) results in
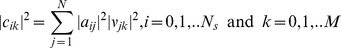
(6)where 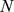 is the number of sources and 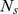 the number of signals 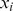 or, in other words, the number of rows of the data matrix X = [x(1) … x(T)]. Since the signals are real the Fourier coefficients equations (4) and (5) must fulfil 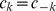
* and 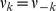
*, 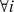. The operator * indicates the conjugate of a complex number. For this reason it is sufficient to solve the demixing problem by considering only the coefficients with index 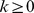. As a consequence of equality (1) also the following equations hold:



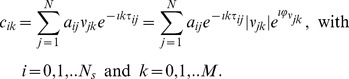
(7)The previous considerations motivate the following iterative algorithm for the identification of the unknown parameters in model (1). After random initialization of the estimated parameters, the following steps are carried out periodically until convergence:

1. Compute the absolute values of the coefficients 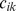 and solve the following equation:

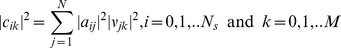
(8)In our implementation we exploit non-negative independent component analysis (ICA) [27] to solve this equation.

1. Initialize 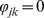 and iterate the following steps:
  - Update the phases of the Fourier coefficients of the sources by solving the following non-linear least square problem
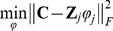
(9)where 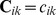, 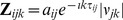, 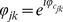 and where we define the result of the operator 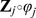 as the matrix of size 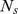 by 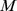 that is obtained by summing over the index 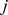 the elements of the matrices Z and *φ* so that 
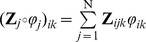
. F indicates the Frobenius norm.
  1. Exploiting the knowledge of the sources 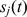 identify the weights 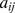 and delays 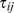 by optimizing the cost function:

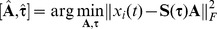
(10)Optimization can be carried out following [28], assuming uncorrelatedness for the sources and independence of the time delays.

The algorithm presented above was inspired by previous work carried out in [24], [26]. Like this previous algorithm, FADA assumes independence of the sources. Opposed to PCA and ICA model, the anechoic mixing model allows for time shifts of the sources. Compared to the our previous algorithm, FADA has much less free parameters that need to be estimated, which makes the algorithm much faster and more robust, and much less prone to get stuck in local minima.

FADA requires to define the number of sources a priori. To compare different models we characterized the approximation quality of mixture model by computing the percentage of variance accounted for (*VAF*) as a function of the number *N* of components. The *VAF* is defined as
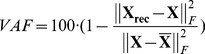
(11)where **X** is the matrix of the recorded trajectories, **X_rec_** is the matrix of the reconstructed data obtained using a certain number of primitives, and 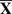 is a matrix of the same size of the matrix **X** and whose rows are equal point by point to the mean values of the corresponding rows of **X,** i.e. the mean of the angular trajectories in the rows of **X**. A simple criterion to determine the relevant number of primitives is to determine the point where the cumulative *VAF* levels of and saturates (‘scree test’). In our study we varied the number *N* of temporal components between 1 and 5. The exact point of change of slope was quantitatively determined by using a linear regression procedure [15], [16], [19], [20], [29]. We computed a series of linear regressions, starting from a regression on the entire cumulative *VAF* curve and progressively removing the smallest value of the number of components from the regression interval. We then computed the mean square residual error of the different regressions and selected the number as optimal for which the corresponding error was smaller than 0.001. For cross-validation purposes, we also extracted *N = 1…5* source components from 80% of the total trials. We then used the components extracted from the training set to reconstruct the trajectories of the remaining trials (test data set) and computed the corresponding *VAF*. To avoid convergence to local minima, we always ran the algorithm 25 different times on the same data set and considered only the solutions that provided the lowest reconstruction errors between original and reconstructed data. The improvements provided by considering time shifts in the generative model underlying FADA and with respect to the performance obtained with standard PCA, which is based on instantaneous mixing models, were assessed. Because of the large differences of magnitude between angular displacements of the upper and the lower-body joints, PCA based on the correlation matrix rather than based on the covariance matrix was performed.

### Control Experiments and Assessment of the Intrinsic System Delays

A first control experiment (3 subjects, who did not took part in the main experiment, 3 males, ages 24±3 years, mass 67±8 kg, height 1.74±0.05 m) was carried to test whether the periods in which the target turned yellow might have had an influence on the result by providing a signal that participants could have used to show predictive behaviour. To preclude such possibility to predict the target ball remained red even during the step cycle preceding the “go” signal.

A second control experiment (3 subjects, who did not took part in the main experiment, 3 males, ages 25±4 years, mass 65±9 kg, height 1.69±0.04 m) was carried to test whether the temporal relationships between the first four foot step events following the “go” signal and the time of reaching the target were dependent on walking speed. The conditions were as in the first control experiment (no yellow phase anticipating the “go” signal) and walking speed was, in two separate experimental sessions, either 0.6 m/s or 1.00 m/s (corresponding to 75% and 125% of the walking velocity in the main experiment). In this control experiment only two triggering events were considered instead of four, the lift and the strike of only the right foot (RHL and RTL).

In the Virtual Reality setup an intrinsic time delay between event detection and stimulus presentation cannot be avoided. Once the foot event occurs, the system requires some time to detect the event, process the information and send back a signal to the VR system to change the colour of the virtual target. It was thus important to estimate the magnitude of this loop delay. For this purpose, we computed for all the trials of each participant the difference between the time of the change of sign of the foot velocity (determined offline by visual inspection of the time course of the velocity) and the time of occurrence of the “go” signal stored in the log files. The average value of these time differences was then taken as an estimate of the delay introduced by the system loop. Following to such a procedure we found an average delay of 38±15 ms. These temporal delays are so small that they do not substantially bias the reported delays, so that we neglected this factor in the interpretation of the data.

### Statistical Analysis

Statistical effects were tested by performing 4×5 (4 foot events and 5 target locations) multivariate analyses of variance (ANOVA), where appropriate. Post-hoc analyses were carried out by using a multiple comparison method (with Tukey–Kramer correction). Student’s t-test was used to test whether the relative time delays between time of pointing and a foot event was significantly different from 0. Student’s t-test was also used to test whether the times of occurrence of specific foot events were statistically different or not. In all cases the level of significance at which the null hypotheses were rejected in the study was set at 1%. A chi-square goodness-of-fit test was always used to test the normality of the delay distributions.

## Results

### General Kinematics

All participants were able to accomplish 100 successful reaching movements (5 ball positions×4 foot events×5 movements per position). However the task requirements made the task quite difficult to accomplish. Participants had indeed to perform, on average, 151±23 movements in total, including the trials that were discarded.

Although four foot events were used to trigger the visual stimuli, one might conclude that only a subset of them is really relevant for coordination, since some of them were temporally quite close (for instance LHS might coincide with RTL and RHS with LTL). In order to rule out this possible confound, we computed the average time differences, as percentage of the total gait cycle duration, and the corresponding standard deviations between the time points of LHS (RHS) and RTL (LTL). We found significant differences of 13±7% for RTL - LHS, and of 13±5% for LTL - RHS. In both cases t-tests revealed that these temporal differences were statistically different from zero (p<0.01), indicating that the four foot events really were appropriately used as trigger signals. Since the results for the left foot events were however very similar to those for the right foot events we report in the following for simplicity only the results for RTL and RHS.

We computed the average values of several kinematic parameters in order to characterize the walking and reaching patterns for the individual participants (Table 1). Although the treadmill was operating at a constant velocity of 0.8 m/s (2.88 km/h) the mean walking velocities in table 1 were typically smaller, with a few exceptions. This indicates that, within the two gait cycles taken into consideration, the subjects tended to shift slightly backwards in the direction of walking. This might be a consequence of a reduction of walking speed that was necessary as preparation of the arm movement. An ANOVA revealed a significant dependence of the mean cycle duration on both target location (p<0.001) and triggering event (p<0.001).

**Table 1:** General kinematic parameters (mean ± SD).

		Mean walking vel.(Km/h)	Mean gait cycleduration (s)	Mean stance phaseduration (% cycle)	Mean reachingduration (% cycle)	Hand peak vel.(Km/h)
		*				
**P1**	**RTL**	2.77±0.18	1.16±0.20	0.57±0.24	1.05±0.38	13.61±3.84
	**RHS**	2.83±0.13	1.20±0.14	0.57±0.20	1.11±0.32	13.78±2.32
					*	
**P2**	**RTL**	2.85±0.11	1.19±0.13	0.61±0.15	1.03±0.28	13.89±3.00
	**RHS**	2.88±0.14	1.18±0.16	0.62±0.04	1.14±0.36	13.30±2.75
			*			
**P3**	**RTL**	2.83±0.10	1.25±0.13	0.61±0.11	0.85±0.21	10.15±2.59
	**RHS**	2.87±0.11	1.23±0.13	0.59±0.18	0.89±0.27	10.15±2.75
						*
**P4**	**RTL**	2.79±0.11	1.15±0.20	0.59±0.18	0.83±0.37	9.31±2.65
	**RHS**	2.79±0.13	1.16±0.14	0.58±0.20	0.90±0.35	8.47±2.46
		*				*
**P5**	**RTL**	2.81±0.12	1.22±0.15	0.61±0.16	0.88±0.22	9.16±2.34
	**RHS**	2.87±0.14	1.24±0.14	0.59±0.15	0.94±0.26	7.55±2.23

**M**ean values ad corresponding standard deviations of walking velocity, gait cycle, stance phase and reaching durations along with the mean hand peak velocity are reported for each target location (P1,…,P5) and right foot triggering events RTL and RHS. Asterisks indicate significant statistical differences between the average values of a kinematic parameter for the trigger events RTL and RHS.

The mean stance phase duration was, however, neither dependent on the target position (p = 0.37) nor on the triggering event (p = 0.62). The mean reaching duration was found to be significantly modulated by both target location (p<<0.001) and foot event (p<<0.001). The durations were higher for the two highest target positions. Similarly, the hand peak velocity depended on both target location (p<<0.001) and foot event (p<0.01). As for the mean reaching duration, the mean hand peak velocities associated with the two highest target positions were significantly higher than those of the other locations. No statistically significant interactions were found between target locations and triggering events, with the only exception of an effect on the mean stance duration (p<0.01).

### Analysis of the Joint Angles

**Figure 2:**
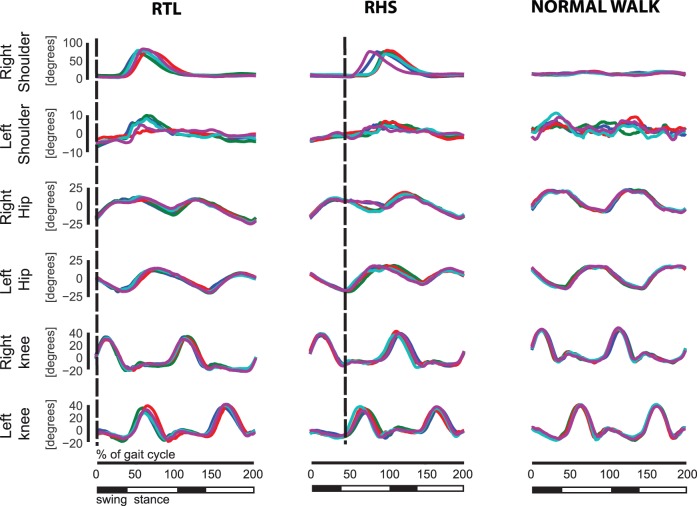
Time course of the main flexion joint angles. Each trace is associated with a single experimental trial. In the first two columns on the left show the results for the right foot events (heel strike and toe lift-off). The vertical dashed lines indicate the time of the “go” signal. Abbreviations at the top of the panels indicate the triggering event (RTL = Right Toe Lift-off, RHS = Right Heel Strike). The last column on the right depicts the evolutions of the joint angles during normal walking.

**Figure 3:**
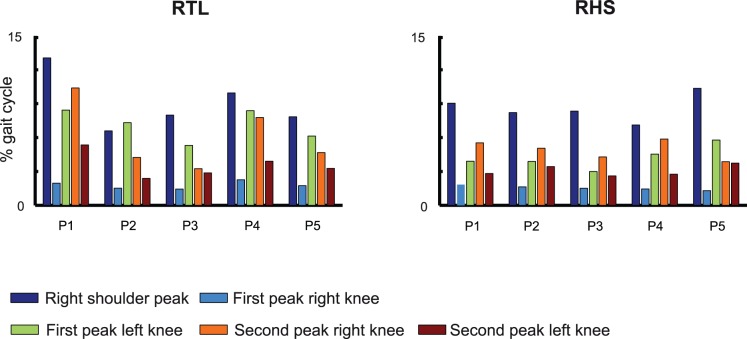
Variability of the joint angles. Standard deviations of the time of occurrence of the peaks for the right shoulder and the four peaks for the knee angles. Abbreviations at the top of each panel refer to the experimental conditions/foot events as in figure 2. Results are reported separately for each target position (P1,…,P5).

Figure 2 shows the reconstructed time courses of six flexion-angles (of both shoulders, hips and knees) from the trials of one typical subject, reaching towards the highest and most leftward target location. Note that we decided to display the angles associated with this position because participants reported it as the most difficult location to reach. The first two columns from the left side of the figure display the temporal evolution of the angles from trials in which the “go” signal was triggered by a specific foot event associated with the right foot. The third column illustrates instead, for comparison, the temporal evolutions of the angles during normal locomotion, when no trigger event occurs. All angles show a stereotypical time course. Within trials associated with the same triggering event the flexion-angles of the right shoulder were always characterized by a discrete, almost bell-shaped angular profile (average peak-to-peak rotation was 96.8±25.8 degrees). The angles of the left shoulder, however, were characterized by much smaller rotations (average peak-to-peak rotation 14.3±5.2 degrees). It is interesting to note that the angles of the left shoulder were always positively correlated with the ones of the right shoulder, even if the bell shape of the left shoulder angles were much less marked. The angles of the hips presented an oscillatory trend, and the ones of the left hips were negatively correlated with those of the right hip. The flexion angles of the knees were instead characterized by very similar temporal profiles and differed only by a temporal shift. Clearly, the peaks of the right shoulder occurred much earlier in condition RTL than in condition RHS, indicating a modulation of the average times of occurrence of such peaks by the time of occurrence of the “go” signals. Even if less clearly defined, the same can be said about the peaks of the left shoulder. Although the temporal evolutions were quite stereotypical across subjects and trials, some variability was noticeable. The most pronounced peaks (figure 2) were those characterizing the evolution of the right shoulder angles as well as of the angles of the right and left knees. We computed the standard deviations, across all the participants, of the times at which these peaks occurred (figure 3), separately for each position of the virtual ball. The most variable peak was, in all conditions, the one of the right shoulder. The least variability was associated with the first peak of the knees that occurred within the two gait cycles taken into consideration. Higher variability was associated with the subsequent peaks of the angles of both right and left knees. This trend was found in all tested experimental conditions (i.e. for trials with different phase relationships between foot contact events and the “go” signal, and for different goal positions). The high standard deviation values for the knee peaks shown in figure 3 suggest a modification of the gait pattern by the pointing movement of the arm. Time intervals between the occurrence of the “go” signal and actual time of target contact were computed for all trials. The analysis of variance of these time intervals revealed that there was a significant dependence of the time intervals on the foot event that triggered the “go” signal (p<<0.001). A post hoc analysis revealed that the average time of reaching relative to RTL (0.52±0.13 s) was significantly different from the reaching times relative to RHS (0.49±0.12 s).

### Coordination between Feet and Reaching Hand

**Figure 4:**
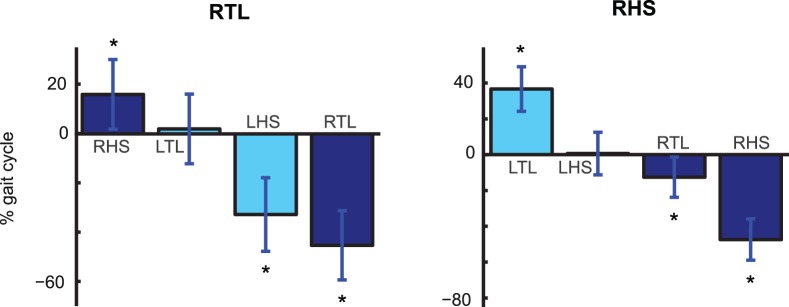
Time coupling between target contact and foot events. Mean values (± SD) of the time interval between time of target contact and the time of occurrence of the first four foot event following the “go” signals. In the figure, RTL stands for Right Toe Lift-off, RHS, Right Heel Strike, LTL, Left Toe Lift-off and LHS for Left Heel Strike. Although the results in the figure refer to trials triggered with kinematic events of the right foot, highly similar findings were obtained for trials triggered by left foot events.

For each trial the intervals between the time of reaching and the time of occurrence of the following four foot events were computed. The average values of these time intervals are shown if figure 4. The means were computed across the trials in which the “go” signal was triggered with kinematic events of the right foot. The figure shows that the time of the reaching was always approximately synchronized with the time of the second foot event following the “go” signal. Statistical analysis revealed that the average time interval between the time of target achievement and the second foot event following the pointing was not significantly different from 0 for both triggering events (p = 0.4 for RTL and p = 0.43 for RHS), indicating perfect synchronization of the two events in two out of four experimental conditions. The same behavioural trend was observed when within-subject analysis was carried out, confirming the synchronization of the reaching time with the second foot step event following the “go” signal.

### Dimensionality Reduction

**Figure 5:**
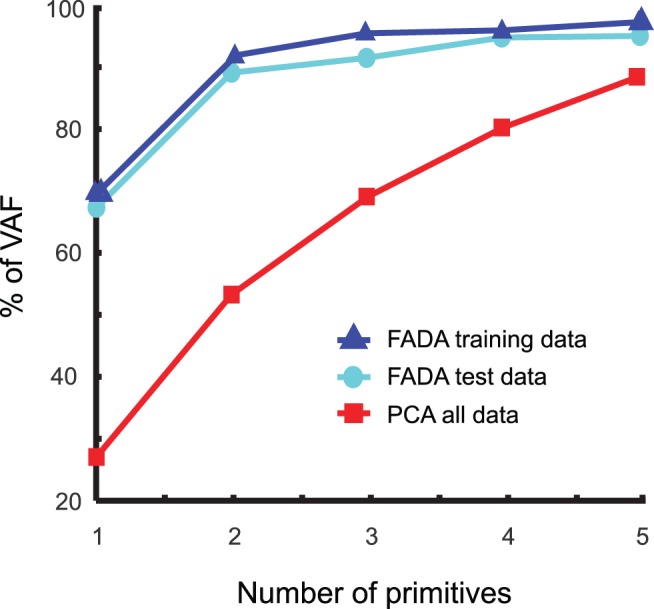
Variance accounted for (*VAF*) as a function of the number of primitives. The red squares refer to the level of reconstruction of the original data obtained with PCA. The blue triangles refer to the *VAF* associated with the reconstruction of the training data with FADA. The cyan circles report the percentage of *VAF* associated with the test data set when it is approximated by superposition of the time shiftable sources learned with FADA applied to the test data set (cross-validation step).

We applied two dimension reduction techniques, PCA and the new FADA algorithm, to a data set that included the flexion-angle trajectories from shoulders, hips and knees from all valid trials in the main experiment. The goal of this analysis was to uncover a potential modular organization of the coordination patterns that are apparent in the kinematics of the shoulder, hips and knees. The two applied unsupervised learning techniques are based on two different generative models, with and without additional time delays for the superpositioned source signals. For both methods we selected the minimum number of primitives (source signals) that was required to account for the major part of the variance of the data, by investigating how approximation quality, measured by the *VAF*, depends on the number of sources in the fitted model (figure 5). Qualitatively, it seems evident that the *VAF* as a function of the number of sources for the FADA suggests that a model with two sources approximates the data in an optimal way. The existence of an “elbow” in the *VAF* curve for *N* = 2 indicates that additional sources capture only very small additional amounts of variance of the data. The *VAF* curve associated with PCA, instead, shows a much more gradual increase, and it appears that even 5 sources are not yet sufficient to provide a good approximation of the data. This qualitative impression is confirmed by the linear regression procedure that we used to quantitatively validate the choice of the number of primitives. Only when applied to the curve relative to FADA the regression procedure could identify the point at which the mean square error dropped below threshold. In the PCA case the criterion did not provide a solution, indicating that a model with less than six sources seem not adequately to capture the data.

**Figure 6:**
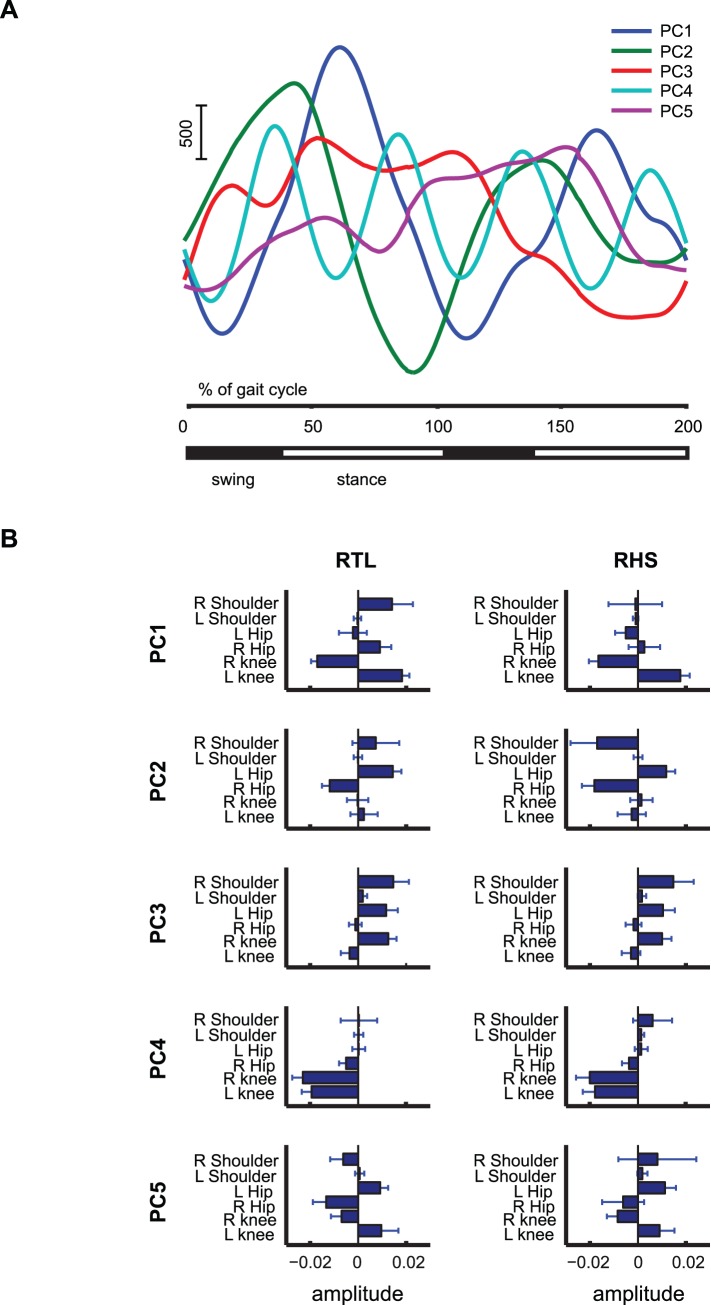
Results of dimensionality reduction with PCA. (**A**) Time functions corresponding to the 5 principal dominant components extracted from the whole joint-angle data set. (**B**) Corresponding weighting coefficients.

The temporal evolutions of the first 5 PCs are shown in figure 6A. The identified PCs were characterized by a complex oscillatory structure. Some components (e.g. PCs 1 and 2) seem to be characterized by similar frequencies of oscillation, but are time-shifted against each other. Also PC3 and PC5 seem similar, but time-shifted against each other. PC4 did not match with any of the other PCs. The large number of significant PCs makes it difficult at first sight to provide a meaningful interpretation.

The weight coefficients corresponding to the five PCs are depicted in figure 6B, separately for the RTL and RHS experimental conditions. The coefficients belonging to the left shoulder and the lower body joints have approximately the same amplitude across the two conditions. The coefficient of the right shoulder is instead much more variable. It has the same amplitude when referring to PC3, approximately the same amplitude in absolute value but opposite sign across condition when referring to PC2 and PC5 and totally different amplitude when scaling PC1 and PC4. These observations illustrate again that the PCA results are quite difficult to interpret and to exploit for getting an intuition about the structure of the underlying coordination patterns.

**Figure 7:**
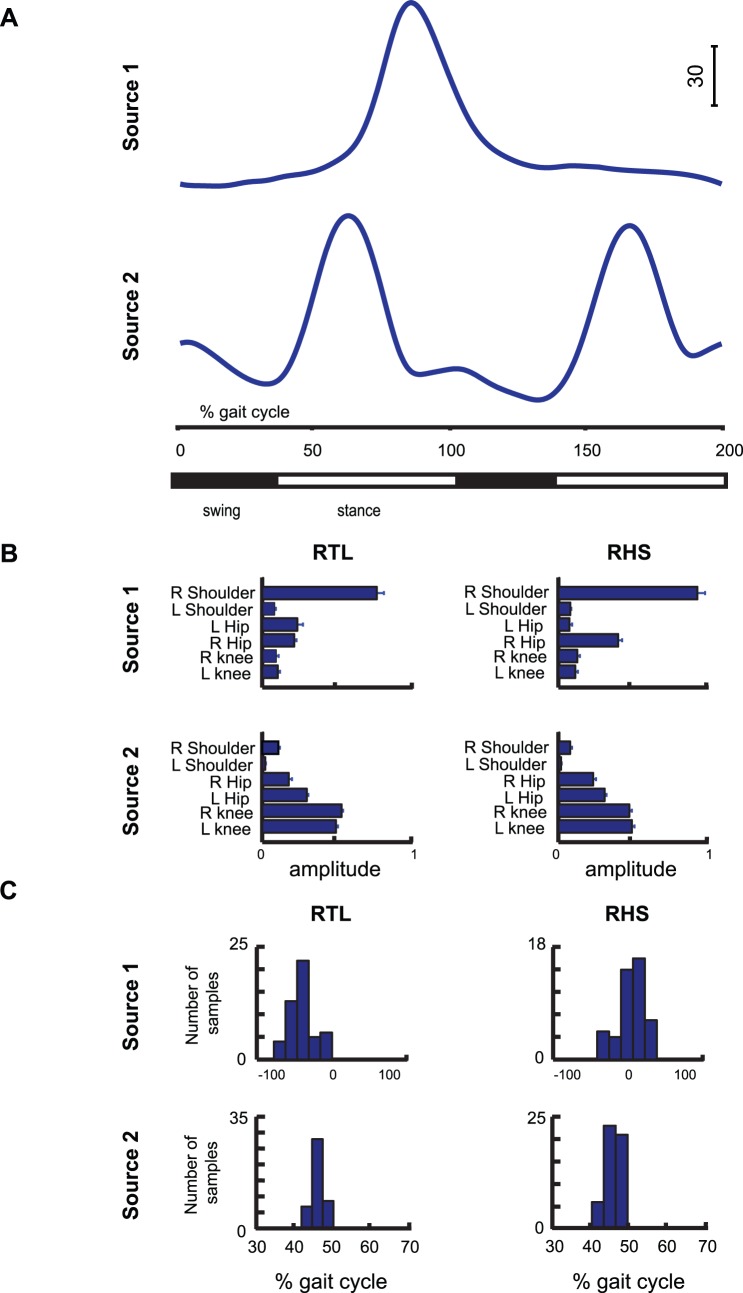
Results of dimension reduction with an anechoic demixing algorithm (FADA). (**A**) Time courses of the two temporal sources extracted with the FADA algorithm, which is based on the generative model (1). (**B**) Average values of the weighting coefficients associated with the two sources. (**C**) Time delays distributions for each source and condition.

The two source functions identified by means of the FADA algorithm are shown in figure 7A. One is a single bump, while the other one is more oscillatory with two distinct peaks. From the comparison of these waveforms with the ones in figure 2 it is straightforward to note that the first primitive seems more related to the right shoulder angle, while the second primitive seems to be related to the evolution of the angles of hips and knees. The relative strength of the effect of each source on a given angle trajectory is provided by the corresponding mixing weights. The mean values of these weights, averaged over all subjects and valid trials with reaches to the most upward and most leftward position are shown in figure 7B. For each source the weights were computed separately for experimental conditions and joint angles. The largest weight coefficient related to the first source was the one of the right shoulder angle, while for the second source there was a progressive increase of the amplitude of the weights when moving from the ones associated with the upper body joints to the ones of the lower body. The two extracted waveforms seem to be task-dependent: one more associated with the reaching discrete sub-task, the second one to the periodic sub-task, i.e. walking. However, this task separation is not complete in the joint space, since both primitives contribute actually to the trajectories of all joints, even if more markedly for some body parts. Although, for instance, the coefficient of the right shoulder is dominant across all experimental conditions, even the other coefficients are not zero. Similarly the weight coefficients for the second source of the shoulders are low, but non-zero. These considerations, made for the weights obtained for the most upward and most leftward reaching position were also valid for the other ball locations. In order to assess this invariance, we computed the average correlation coefficients between the weight vectors associated with different ball positions. We found that, on average, R = 0.98±0.01. A further proof of the strong relationship between the extracted sources of figure 7A and the two main movement sub-tasks (the periodic and discrete one) is provided by the distribution of the temporal delays that correspond to the two sources. Figure 7C illustrates, for each source and experimental condition, the distribution of the delays associated with all trials accomplished to reach the most upward and most leftward position. The delays are always distributed about well-defined average values, with approximately Gaussian distributions. Note that these findings are coherent with figure 2, where the peaks of the right shoulder angle occur earlier in the data for the triggering event RTL than for RHS. Also a post-hoc analysis for the delays of the second source did not reveal statistical differences between the average delays for the conditions RTL and RHS. These results are coherent with figure 2.

The findings presented above were further confirmed through a cross validation procedure. Figure 5 shows the *VAF* measure for a training data set, used for the fitting of the generative model (1), and for a test data set that was not used for this fitting. The regression procedure confirmed that 2 sources provide a good level of reconstruction of the data. Moreover, the two sources extracted from the training data set were found to be very similar to those in Fig. 7A (average correlation coefficient R = 0.998±0.001, after that the corresponding sources were re-aligned according to their maximum peaks).

In summary, we have shown in this section that the temporal evolution of the task-relevant joint angle trajectories can be well described by a modular organization. Comparing PCA as classical approach that is based on an instantaneous mixture model and the novel FADA algorithm, which fits an anechoic mixture model, we found that the FADA algorithm recovers a nicely structured model with only two source components. Contrasting with this result, the PCA model requires a large number of components that seem not to have a clear intuitive interpretation, and results in a weight distribution that is quite complex and difficult to analyse in dependence of the experimental conditions. The two source components extracted by the FADA algorithm map intuitively onto coordination patterns of the arm and the lower body that seem to be useful to accomplish the rhythmic subtask (walking) and discrete sub-task (reaching).

### Control Experiments

We described above a dependency of the target reaching time on the foot step event triggering the “go” signal. To test whether such a dependency might be caused by some anticipatory mechanisms induced by the fact that the target balls turned yellow one gait cycle before the “go” signal, we asked to three control subjects to perform the same tasks without presenting a colour switch to yellow before the “go” signal. The ball turned directly from red to green at the time of occurrence of the triggering event. Analysis of variance revealed that even in this case the reaching time was significantly dependent on the foot step event triggering the “go” signal (p<<0.001). Post hoc analysis showed that the average time of reaching was that relative to the triggering event RTL was 0.50±0.04 s and it was 0.48±0.04 s for RHS. The control experiment therefore confirmed the results reported for the main experiment, and thus proves that the phase with the yellow ball preceding the “go” signal did not play any role in the modulation of the timing of reaching.

**Figure 8:**
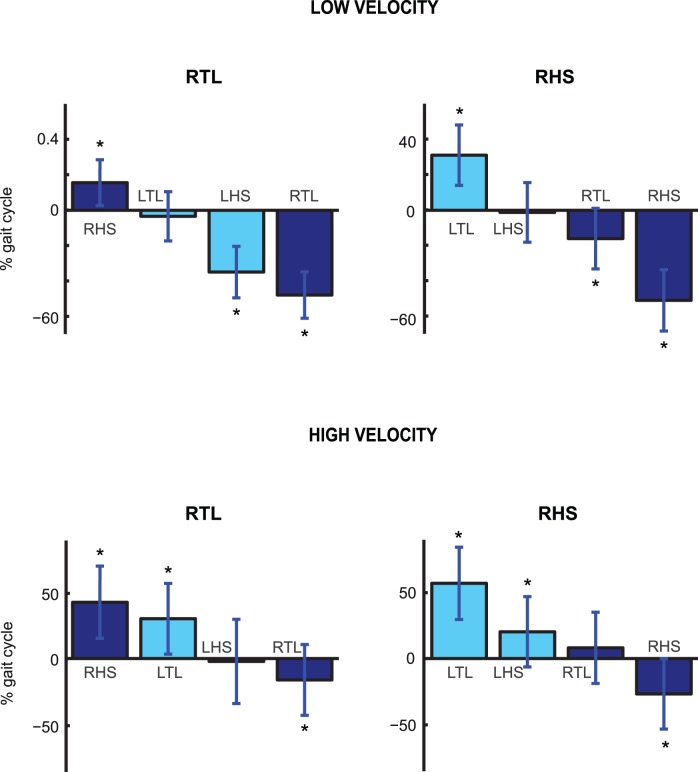
Time coupling between target achievements and foot events (control experiment). Mean values (± SD) of the time interval between time of target contact and the time of occurrence of the first four foot events following the “go” signals. The two top panels show the results for the conditions RTL and RHS when the treadmill was operating at slow velocity (75% of the velocity in the main experiment). Bottom panels show the data collected for same conditions with the treadmill operating at high speed (125% of the speed in the main experiment).

We reported above and in figure 4 a strong time coupling between the times at which the targets were reached and the times of occurrence of the second footstep events following the “go” signal. In order to assess whether this temporal coordination is just an artefact because the time between “go” signal and the reaching of the goal was close to the duration of a half gait cycle, we realized a control experiment with three participants that were asked to walk on the treadmill at two different velocities, corresponding tom 75% respectively 125% of the walking velocity in the main experiment. The results reported in figure 8 (top panels) show that in the case of low velocity the time of the reaching of the target was not statistically different (p>0.01) from the time of occurrence of the lift of the left foot when the “go” signal was triggered with the lift of the right foot. Similarly, when the “go” signal followed a strike of the right foot, the time of target achievement occurred, on average, simultaneously with the strike of the left foot (p>0.01). These results are in agreement with those in figure 4. When the velocity of the treadmill was increased (and thus the average gait cycle duration reduced), however, the time at which the target was reached was still synchronous with a specific footstep event (figure 8, bottom panels). But in this case it was the third and not the second event following the “go” signal (p>0.01 in the RTL and RHS conditions). A within-subject analysis confirmed that each participant tended to synchronise the time of reaching with the second foot step event for the two conditions that were run at low velocity. For the high velocity condition one of the three participants showed a behaviour that differed from the other two. All participants synchronized the reaching with a specific foot-step event. However, the number of the event varied from subject to subject. (One synchronized with the second, one with the third, and one with the fourth.).

## Discussion

In this study we investigated the kinematic patterns of coordination that underlie complex whole-body movements, which combine arm pointing to a virtual target in space and walking. Consistent with a coordination of specific movement primitives for locomotion and goal-directed reaching, we found a modification of the walking pattern subsequent to the arm movement, as well as a modulation of the arm movement by the foot events triggering the time of reaching. In particular, we found a strong temporal coupling between the time when the hand reached the target and the time of the heel strike and the toe lift. Furthermore, by applying a new statistical technique for the extraction of movement primitives from the angle trajectories by anechoic demixing (using the newly developed FADA algorithm) we found the existence of two main kinematic components that were related to the walking and reaching parts of the coordinated movement. In the following we discuss the possible origins of the observed coordination patterns between reaching and locomotion. In addition, we briefly discuss the differences between the FADA technique and traditional demixing approaches, such as PCA.

### Control Mechanisms of Pointing during Locomotion

**Figure 9:**
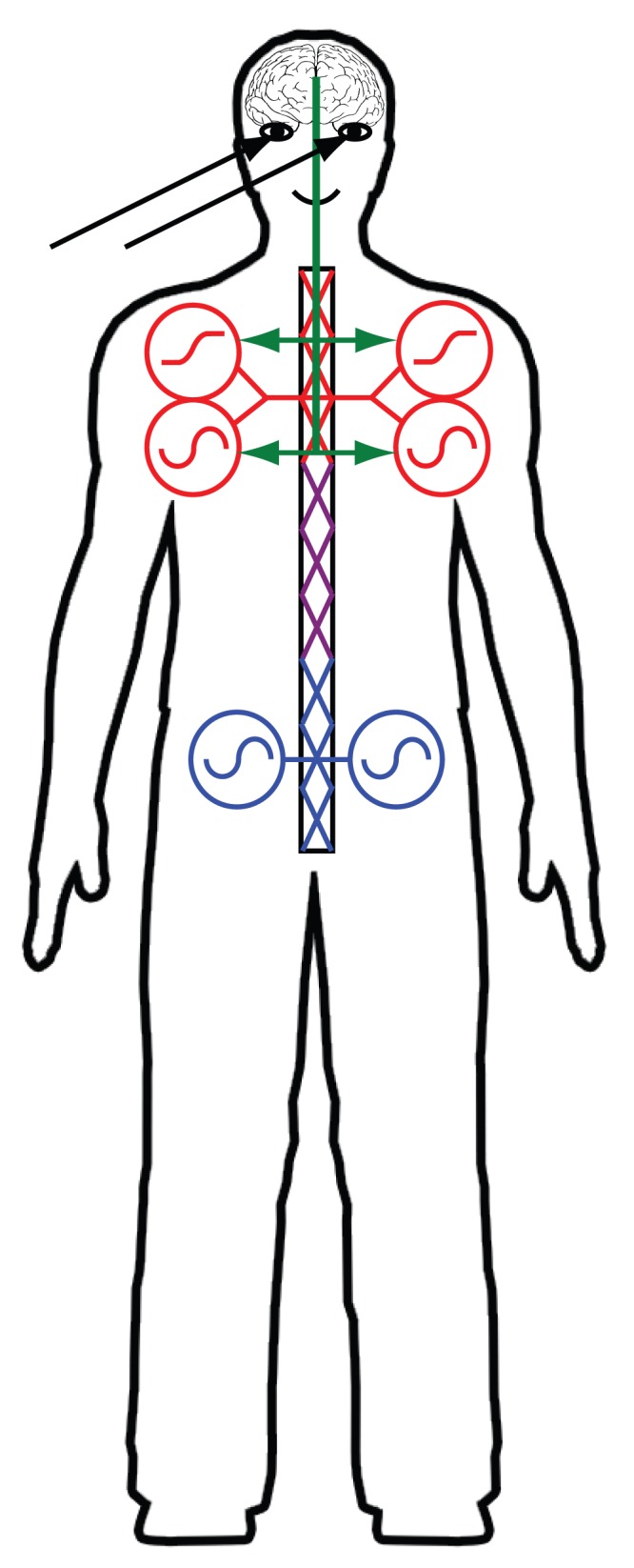
Schematic illustration of a possible control architecture for goal-directed reaching movements while walking. During normal locomotion CPGs producing periodic signals (sine waves within the symbols) are responsible for the periodic behaviour of both upper and lower limbs In addition, another set of CPGs might also be involved in the control of discrete movements, e.g. of the arm (ramp-like function in the CPG symbol). All are coupled to each other via neural networks in the spinal cord. Their activity can also be modulated by sensory feedback (not illustrated in the figure). After the detection of the “go” signal through the visual system (black lines), a descending command is generated by cortical areas (green lines) and sent spinal segments through descending pathways. This command activates the CPGs for the generation of the discrete arm movement. It also might deactivate or reduce the influence of the CPGs that generate the periodic movement for the upper limbs. How such a deactivation process might work is still an open question. Thus, in the figure the descending command on the CPGs of arm was schematised. The violet lines in the sketch of the spinal cord indicate s network of neurons that couples the CPGs of arms and legs.

It is well-known that arm reaching from a standing position elicits a set of postural adjustments, affecting the lower body joints in order to counterbalance the perturbations introduced by the motion of the upper limb [30], [31], [32], [33]. In this case, the postural task (balance adjustment) seems to be induced by the reaching task, with the aim to guarantee body equilibrium in spite of the destabilizing effects caused by the perturbations introduced by the voluntary movement. Our results show that, when this simple postural task is replaced by a dynamical one (walking), the rhythmic pattern associated with the lower limbs is modified as consequence of the concurrent reaching task. This is in agreement with the mechanisms that were found regulating the interaction between voluntary arm movements and posture, and also with the observations in other studies on the interaction between arm reaching and locomotion [4], [5], [6], [7], [8]. In our experimental paradigm we had the possibility to control with a high level of precision the time of the “go” signal in relationship to different foot events during the gait cycle. This made possible to show that the reaching task is also to some extent modulated by temporal structure of the walking task. We therefore found a mutual interaction between reaching and locomotion. The results of our study motivate interesting ideas regarding the planning mechanisms that are required for accomplishing our experimental tasks. They support in particular the hypothesis that reaching and walking are integrated within a coherent motor plan, instead of being planned completely separately. The dependency of the duration of the time interval between the “go” signal and the target contact on the type of triggering event suggests, first of all, that the control of the reaching movement, which potentially involves also internal models represented in higher brain structures, is dependent on the states of pattern generators that are involved in the control of locomotion. Specifically, we found in the main experiment that the time of target contact was always strongly coupled with the second foot event occurring after the “go” signal. Thus, for instance, if the lift of the right toe was the triggering event, the instant of maximum arm extension at which the target was reached coincided with the time of occurrence of the next lift of the left toe (time of maximum extension of on the two legs). This type of coordination pattern was independent of the gait cycle time. We do not have yet a direct explanation that can account for this strong time coupling, which is also described by the features characterizing the values assumed by the weights and the distributions of the delays associated with the two components illustrated in figure 7. It can surely be hypothesized that such a coupling might be partly due to biomechanical reasons, such as the need to maintain dynamical stability during locomotion in presence of the arm movement. Another explanation can be derived from the neural structures in the spinal cord that are involved in the control of arm and leg movements during walking (see [34] for a detailed review). Although in humans it has been always harder to provide evidence for the existence of central pattern generators (CPGs) than in animals, some data has become available that such circuits, which might be partially localized in the spinal cord and which generate and shape the patterns of the bursts of motoneurons [35], [36], also exist in humans. In addition, since walking normally is associated with coordinated movements of arms and legs is seems likely that such networks of pattern generator affect upper and lower limbs during locomotion. The CPGs controlling arms and leg likely are coupled with each other, and their activities are additionally modulated by supraspinal inputs and sensory feedback. In light of observations provided by a multitude of previous studies on animals and humans, and of the results of our study, we agree with a hypothesis suggested by Georgopoulos and Grillner [1] that the reaching behaviour during walking might arise from the interaction of voluntary motor commands originating in the supraspinal regions with the unconscious control of the CPG networks in the spinal cord, which might control the rhythmic movement of arms and legs during locomotion, but which may also be involved also in the control of non-rhythmic behaviours. A sketch of such an architecture for motor control organization is illustrated in figure 9. In this framework the CPGs of arms and legs are responsible for the generation of the rhythmic oscillations of the limbs during walking. During the execution of the reaching task, however, a voluntary motor command is sent from the cortical regions to the spinal cord through descending pathways. The role of this command could be exerting (or not) an inhibitory action on the rhythmic activity provided by the CPGs of the arms while activating concurrently another spinal network, or primitive, that mediates the control of the arm movement for reaching. Although no direct physiological evidence for such a hierarchical control structure has been provided, computational models have been developed that support the efficiency of such control structures, and which have allowed to synthesize realistic complex body movements involving periodic movement primitives as well as discrete dynamical primitives [25], [37], [38], [39]. The dependency of the duration of the time interval between the “go” signal and the time of target contact on the triggering foot event could reflect an interaction between the descending central command and inputs from the CPG network that controls the locomotion, potentially optimizing the reaching behaviour by ensuring target contact during specific phases of the gait cycle. In addition, a phase-dependent modulation of reflexes in the might contribute to the modulation of the gait pattern in dependence of such top-down control signals for reaching. It is well established that reflexes are adaptive and dynamic, as they both depend on intentions and on the activation states of the muscles [40]. Reflex gains might thus change dynamically during locomotion and might be modulated by additional higher-level commands that optimize the stability of the walking behaviour for the concurrent reaching action.

The neurophysiological structure potentially underlying the generation of arm movements during locomotion that we have proposed above is compatible with a number of evolutionary considerations. Bipedal human locomotion is the result of a phylogenetic process that has brought man to walk from a quadrupedal stance to an erect position. It has however been shown [41] that bipedal locomotion still shares common spinal neural control mechanisms with quadrupedal locomotion, such as the coordination of upper and lower limb movements. In accordance to this, we have found that the periodic component in figure 7A is distributed across all body joints (see weight coefficients in figure 7B). At the same time, switching from quadrupedal to bipedal locomotion in evolution has allowed to use of the arms for additional motor tasks, such as grasping and object manipulation. Such tasks rely, more than locomotion, on the use of supraspinal and cortical structures. This has resulted in a superposition of older systems, like CPGs for the generation of rhythmic patterns from locomotion, by the control or modulation through higher structures that are responsible for the control of voluntary and goal-directed movements. In addition, the control of such goal-directed movements might also include spinal components, e.g. in terms of reflex loops. This is illustrated in figure 7 by the discrete kinematic component, which affects mainly the joints of the right shoulder.

### Decomposition of the Coordinative Kinematic Patterns in Rhythmic and Discrete Components

An important result of our study was that the FADA algorithm applied to the trajectories of the most significant joint angles revealed only two kinematic components, which accounted with high accuracy for the variance of the relevant joint angle trajectories, and which were easily interpretable. One component was found to carry more information about the kinematic co-variation of the lower body joints, and seems to be related to the rhythmic walking task. The second component was found to characterize mainly the angular displacement of the right shoulder, and seems thus to be related to the discrete pointing sub-task. Contrasting with this result, the application of PCA that relies on an instantaneous generative mixing model (where sources cannot be time-shifted), to the same data resulted in rather ambiguous results. It appears that even 5 PCs were not sufficient to capture the variability of the data. Likely, this high number of sources is a consequence of the inadequacy of the underlying generative model, which cannot model temporal delays appropriately and requires the introduction of new sources to account for signals that are time shifted against each other. The observed much higher compactness of anechoic generative models matches our previous experience with many other kinematic data sets [22], [26]. As consequence of the inadequacy of the generative model, PCA requires the introduction of a large number of sources and mixture weights, resulting in the problem that the parameters of such models are difficult to estimate robustly from limited amounts of data, and even more difficult to interpret. This complexity was reflected in the complex shape of the recovered source functions (figure 6A) and the very complex dependence of the mixture weights on the different experimental conditions (figure 6B).

Unsupervised learning techniques for reduction of dimensionality have been widely applied for the study of movement coordination in the field of movement science. In previous studies using these techniques, however, purely discrete [14], [15], [16], [17] or rhythmic [22] movements have been investigated, with very few exceptions for hybrid behaviours [42]. Our study is, to our knowledge, the first that has applied such techniques to investigate the kinematic coordination regulating a combination of rhythmic and discrete movements. For this case we could show that using improved generative models for data analysis might help substantially to uncover the structure of underlying coordination patterns, and potentially of underlying control modules.

## Conclusions

We have studied the kinematic patterns of joint co-variation associated with a motor task requiring arm pointing while walking. We found a mutual interaction between walking (rhythmic) and arm reaching (discrete task). The results suggest that, at execution level, the two motor tasks may be regulated by the interaction of two different control modules, whereas they seem, at planning level, to be integrated in a single motor plan that aims at a synchronization of the discrete goal-directed movement with specific events of the rhythmic movement, and a modulation of walking, potentially optimizing gait stability under the influence of the additional discrete behaviour.
